# Social Media and Clinical Trials in Pediatric Disease: Musings From the Bench

**DOI:** 10.3389/fgene.2021.734691

**Published:** 2021-10-04

**Authors:** Leslie Nelson, Susan T. Iannaccone

**Affiliations:** ^1^Department of Physical Therapy, University of Texas Southwestern Medical Center, Dallas, TX, United States; ^2^Department of Pediatrics, University of Texas Southwestern Medical Center, Dallas, TX, United States; ^3^Department of Neurology, University of Texas Southwestern Medical Center, Dallas, TX, United States

**Keywords:** social media, social networking, clinical trials, clinical research, neuromuscular disease

## Abstract

The use of social media in clinical trials, for recruiting and retention as well as for collecting data, has become increasingly common. However, little has been documented in respect to the guidelines for its use and the possible effects it may have on clinical trials. In this review, we provide an overview of the guidance that has been published and muse the pros and cons of the use of social media in trials for rare disease.

## Introduction

“There’s a revolution going on.” It is a revolution in the design and implementation of clinical trials (CTs). This revolution seems most evident in research that affects patient populations that were long left out of the process: those affected by childhood and rare diseases. Prior to the last 2 decades, clinical trials (CTs) in children were unusual outside the field of oncology. Trials were limited due to the challenge of providing for an ethical informed consent by proxy when the subject is a minor. Secondly, diseases of childhood tend to be rare. Clinician scientists could only dream of organizing trials for patients with rare disease, fearing the huge challenge of meeting rigors of “statistical power” with a small pool of patients. In this new age, we are seeing CTs with n of one. What happened and is it good science?

Those of us in the field of pediatric neuromuscular disease have been privy to watch the revolution unfold. It started with the “common” rare disease Duchenne muscular dystrophy (DMD), resulted in the near cure of another “common” rare disease spinal muscular atrophy (SMA) and may bring about miraculous recovery for several ultra-rare neurodegenerative diseases in the near future. We suggest there are three important factors contributing to this revolution:

1. The expansion of patient advocacy that began during the AIDS epidemic of the 1980s
2. The rise of private foundations who finance basic science research into “their” disease
3. Recognition by clinician scientists that patients are a permanent part of the research team.

Along the way, patients (or their guardians in the case of children) have participated in human research by sitting on Institutional Review Boards (IRBs), in drug development by consulting with basic scientists in academics and industry, and in clinical trial design by presiding over symposia and advisory boards. The rise of social media (SM) has allowed researchers a new way to engage with a wide audience and yet also target specific populations based on their online interests, group memberships, and online behaviors. Networking sites are increasingly being used to recruit subjects or to provide forums for participants to communicate with one another. Needless to say, SM has become an integral part of clinical trials, potentially increasing recruitment and retention, but at the same time, raising the risk that scientific rigor might be diluted when patients or their advocates are actively participating. Here is some of what has been written in recent years with regard to this question.

### Roles of Social Media in Clinical Research

There are several ways in which SM can be used in clinical research: recruitment, retention, tracking, and follow-up of subjects. Recruitment and retention in rare disease is challenging and is a critical roadblock for drug development. Limited patient enrollment is the primary cause of missed clinical trial deadlines observed in clinical trials, resulting in delayed availability of potentially life-saving therapies (El Mouelhi, 2016). Recruitment can be complicated due to the small number of available patients and the fact that diagnosis may be difficult, allowing for cases to go unrecognized. Retention may be limited due to significant disease burden endured by patients and caregivers. Complex care is often associated with rare disease and specialty centers available for patients are geographically sparse, adding to burden on patients and caregivers (DeWard et al., 2014). The value that SM may offer in recruitment and retention could be significant. Social networking provides the opportunity for sharing research opportunities with a vast audience that cannot be reached with traditional means of recruiting. It may even stimulate retention by engaging patients on a level that cannot be achieved with traditional clinical interactions as it may spark interest in the research process.

Separate from the use of SM as a recruitment and retention tool, its use as a platform for the research itself may be of benefit in rare disease (Gelinas et al., 2017). A study of patients with acute flaccid myelitis (AFM) utilized SM to collect data and characterize outcomes of its subjects (Bove et al., 2020). Trajectories of these patients could be successfully tracked and reported. Beyond data collection, SM helped counter disparities for these patients and caregivers. In the AFM population, with a scarcity of clinical expertise or information for families, the SM group facilitated important needs by allowing rapid international dissemination of information, integrating information so that those less familiar with the disease could access pertinent information, and even improved quality of care as health professionals adjusted their approach to patient care based on lessons learned from the patients and caregivers.

### Ethical Considerations and Guidelines

It seems that everyone recognizes that there are ethical considerations for using SM but few have laid out specific guidelines. Aside from IRB approval for posting advertisements for recruitment, few investigators indicate exactly what guidelines are for the use of SM. In 2019, Gelinas suggested considerations for the use of SM in clinical trials (Gelinas and Bierer, 2019). Industry sponsors, when establishing a networking page, should ensure the identity of participants is not shared with other group members or the public or that participants consent to their identity being disclosed. When working with minors and their guardians, this becomes especially problematic. Study participants and trial integrity must be protected. Lynch, in 2018, set forth considerations to ensure this protection. First, and foremost, assessment of how use of SM may be problematic in a potential study must be conducted. Collaboration should also include participants and their perspectives. Plans for education for the participants on the potential negative effects of SM on trials as well as information on the ethical responsibility that participants may take on when consenting for a trial. Additionally, alternative communication options may preempt problematic use of SM. An example of this may be offering moderated discussion groups where participants could gain answers in real-time to their questions (Lynch et al., 2018). We found that at our own institution there are no specific requirements for researchers engaging with subjects online (other than restrictions offered by the Health Insurance Portability and Accountability Act (HIPAA). Out of 12 industry sponsored CTs in our own division, none had included any language in the informed consent form (ICF) to guide or limit subjects’ use of SM while participating in the trial (It is important to point out that use of SM to support a CT is different from conducting a study on the internet).

A scoping review from 2018 also found a dearth of guidelines (Hokke et al., 2018). Besides a search of academic peer reviewed publications, Hokke et al. included “grey literature,” sources from institutions and organizations such as committee reviews and theses. This is a remarkable report that should serve as an excellent resource for all clinical trialists. They found five organizations (Table 1) who had published guidelines for internet-based research involving minors; again, this is not the same as using SM to augment CT enrollment/retention. They further cited guidelines from nine universities (Table 2) that covered internet-based research and SM in pediatric CTs and added a few more after their manuscript was accepted. These currently available guidelines offer general advice to aid in ethical decision making when internet research is being conducted. It is noted that they will continue to evolve as the internet evolves and as new issues emerge with development of new online tools. This group followed up their review by conducting a survey in Australia of clinical researchers and members of human research ethics committees, 401 people in all. Some participants had a dual role, meaning they were investigators and sat on ethics committees. They found that only 10% of participants overall had received any training in the specific use of SM in human research. Further, 93% indicated they would benefit from such training. The authors concluded that there is an unmet need among researchers and ethicists and encourage all of us to discuss our experiences using SM. There is a need for education but where that training might be found or in what form is not clear. And how would we evaluate the quality of any SM “expert” who might serve as consultant to the ethics committees?

**Table 1:** Organizations with published guidelines for internet-based research involving minors.

Association of Internet Researchers (Ess, 2002)
British Psychological Society (British Psychological Society, 2013)
Secretary’s Advisory Committee on Human Research Protections (Secretary’s Advisory Committee on Human Research Protections, 2013)
National Committee for Research Ethics in the Social Sciences and the Humanities (Lundh and Ess, 2003)
Carlton Connect Initiative (Clark et al., 2015)

**Table 2:** Universities with published guidelines for internet-based research and social media in pediatric clinical trials.

Florida Atlantic University	https://www.fau.edu/research-admin/research-integrity/research-data/
Pennsylvania State University	https://www.research.psu.edu/irb/policies/guideline10
Queensland University of Technology	https://my.uq.edu.au/information-and-services/information-technology/cyber-security/cyber-security-uq/cyber-security-guidelines-researchers
University of California, Berkeley	http://cphs.berkeley.edu/internet_research.pdf
University of Connecticut	https://ovpr.uconn.edu/services/rics/irb/data-security-guidance-for-human-subjects-research/
University of Rochester	https:/www.rochester.edu/ohsp/documents/ohsp/pdf/policiesAndGuidance/Guideline_for_internet_based_research.pdf
Webster University	https://www.legacy.webster.edu/irb/policy/recommendations_for_use_of_online_surveys.html

On the other hand, some have taken the attitude that no such specific approach is necessary. In one document from the Harvard Catalyst Regulatory Foundations Ethics and Law Program,^1^ the authors state “Social media recruitment is subject to the same regulatory and ethical norms as traditional recruitment, including the requirements of prospective review and approval, compliance with all applicable federal and state laws, fair and equitable subject selection, respect for the privacy and other interests of potential participants, sensitivity to the norms and values of different communities, and consideration for the impacts of different recruitment techniques on public trust in the research enterprise.” In the next 20 pages, they explore how SM presents unique challenges to clinical researchers. They present several case scenarios and review what they consider to be the most ethical approach. This document is meant to be educational and could be an excellent resource for members of research and ethics teams.

## Discussion

We are left with a gap in education regarding the ethics/advisability of using SM for retention and tracking of research subjects. In particular, how can or should we prevent subjects from interfering with scientific objectivity? One of the big risks of SM for CT participants is that discussion among themselves could break the blind or affect retention by influencing subjects to drop out. Online discussions between participants may compromise trial integrity when shared information may inform which study participants may be receiving placebo. Sharing of side effects potentially could influence study results (Ledford, 2018). Commercial influence and exposure to information may bias participants. There is also the potential impact on participants from misinformation that may be shared on SM or even the possibility that information could be transformed incorrectly due to translation that occurs on the networking site. On the other hand, social networking may provide potential participants access to information that may clarify study related issues allowing them to be better informed and armed with information when approaching study investigators with study related questions (Glickman et al., 2012). In addition to the potential influence of SM on participants, researchers themselves may be biased through their interactions *via* SM with patients participating in a clinical trial. Reports that patients may post may bias a researcher in the effects or current status of a study participant that might impact future assessments, especially with more subjective outcomes and assessments.

At this point, it seems the clinical research community should be considering more discussion and investigation of these issues. The use of SM will not diminish in the future and as researchers, we must learn to use SM appropriately. The benefits of SM in rare disease may be many, including the ability to communicate with participants and patients world-wide and across diverse populations. The potential negative consequences of SM, including privacy concerns and impact on trial conduct, cannot be ignored. Education for investigators, participants, patient advocacy groups, and industry on SM would be a good start toward best practices. IRBs should play a part in the discussion as well.

## Author Contributions

SI and LN contributed to conceptualization, data curation and literature review, writing original draft, and review and editing. All authors contributed to the article and approved the submitted version.

## Conflict of Interest

The authors declare that the research was conducted in the absence of any commercial or financial relationships that could be construed as a potential conflict of interest.

## Publisher’s Note

All claims expressed in this article are solely those of the authors and do not necessarily represent those of their affiliated organizations, or those of the publisher, the editors and the reviewers. Any product that may be evaluated in this article, or claim that may be made by its manufacturer, is not guaranteed or endorsed by the publisher.

## Footnotes

^1^ https://catalyst.harvard.edu/wpcontent/uploads/regulatory/Social_Media_Guidance.pdf

## References

[ref1] Bove R. Rowels W. Carleton M. Olivera E. Sheehan M. Werdal H.. (2020). Unmet needs in the evaluation, treatment, and recovery for 167 children affected by acute flaccid myelitis reported by parents through social media. Pediatr. Neurol. 102, 20–27. doi: 10.1016/j.pediatrneurol.2019.08.009, PMID: 31630913

[ref2] British Psychological Society (2013). Ethics Guidelines for Internet-Mediated Research. Leicester: British Psychological Society.

[ref3] Clark K. Duckham M. Guillemin M. Hunter A. McVernon J. O’Keefe C.. (2015). Guidelines for the Ethical Use of Digital Data in Human Research. Melbourne: Carlton Connect Initiatives Fund.

[ref4] DeWard S. J. Wilson A. Bausell H. Volz A. S. Mooney K. (2014). Practical aspects of recruitment and retention in clinical trials of rare genetic diseases: The phenylketonuria (PKU) experience. J. Genet. Couns. 23, 20–28. doi: 10.1007/s10897-013-9642-y, PMID: 24014152

[ref5] El Mouelhi M. (2016). Drug development and challenges for neuromuscular clinical trials. J. Mol. Neurosci. 58, 374–378. doi: 10.1007/s12031-015-0700-9, PMID: 26691331

[ref6] Ess C. (2002). Ethical Decision-Making and Internet Research: Recommendations From the AoIR Ethics Working Committee. USA: Association of Internet Researchers.

[ref7] Gelinas L. Bierer B. E. (2019). Social media as an ethical tool for retention in clinical trials. Am. J. Bioeth. 19, 62–64. doi: 10.1080/15265161.2019.1602425, PMID: 31135316

[ref8] Gelinas L. Pierce R. Winkler S. Cohen I. G. Lynch H. F. Bierer B. E. (2017). Using social media as a research recruitment tool: ethical issues and recommendations. Am. J. Bioeth. 17, 3–14. doi: 10.1080/15265161.2016.1276644, PMID: 28207365 PMC5324729

[ref9] Glickman S. W. Galhenage S. McNair L. Barber Z. Ptel K. Schulman K. A.. (2012). The potential influence of internet-based social networking on the conduct of clinical research studies. J. Empir. Res. Hum. Res. Ethics 7, 71–80. doi: 10.1525/jer.2012.7.1.71, PMID: 22378136

[ref10] Hokke S. Hackworth N. J. Quin N. Bennetts S. K. Win H. Y. Nicholson J. M.. (2018). Ethical issues in using the internet to engage participants in family and child research: a scoping review. PLoS One 13:e0204572. doi: 10.1371/journal.pone.0204572, PMID: 30261041 PMC6160098

[ref11] Ledford H. (2018). A question of control: clinical-trial participants and their carers are gaining influence over how experiments are run. As they take to social media, that could make things messy for the science. Nature 563, 312–315. doi: 10.1038/d41586-018-07351-8, PMID: 30425372

[ref12] Lundh L. G. Ess C. (2003). Research Ethics Guidelines for Internet Research. Norway: National Committee for Research Ethics in the Social Sciences and the Humanities.

[ref13] Lynch H. F. Largent E. A. Joffe S. DeMichele A. M. (2018). Protecting clinical trial participants and study integrity in the age of social media. Cancer 124, 4610–4617. doi: 10.1002/cncr.31748, PMID: 30329153

[ref14] Secretary’s Advisory Committee on Human Research Protections (2013). Considerations and Recommendations Concerning Internet Research and Human Subjects Research Regulations, With Revisions. United States Department of Health and Human Services.

